# Health co-benefits and risks of public health adaptation strategies to climate change: a review of current literature

**DOI:** 10.1007/s00038-012-0422-5

**Published:** 2012-10-31

**Authors:** June J. Cheng, Peter Berry

**Affiliations:** 1 Public Health and Preventive Medicine Program, Department of Clinical Epidemiology and Biostatistics, McMaster University, 1280 Main Street West, HSC2C2, Hamilton, ON L8S 4K1 Canada; 2 Climate Change and Health Office, Health Canada/Santé Canada, 269 Laurier Avenue West, Room 9-062, Postal Locator: 4909C, Ottawa, ON K1A 0K9 Canada

**Keywords:** Climate change adaptation, Health co-benefits, Health risks

## Abstract

**Objectives:**

Many public health adaptation strategies have been identified in response to climate change. This report reviews current literature on health co-benefits and risks of these strategies to gain a better understanding of how they may affect health.

**Methods:**

A literature review was conducted electronically using English language literature from January 2000 to March 2012. Of 812 articles identified, 22 peer-reviewed articles that directly addressed health co-benefits or risks of adaptation were included in the review.

**Results:**

The co-benefits and risks identified in the literature most commonly relate to improvements in health associated with adaptation actions that affect social capital and urban design. Health co-benefits of improvements in social capital have positive influences on mental health, independently of other determinants. Risks included reinforcing existing misconceptions regarding health. Health co-benefits of urban design strategies included reduced obesity, cardiovascular disease and improved mental health through increased physical activity, cooling spaces (e.g., shaded areas), and social connectivity. Risks included pollen allergies with increased urban green space, and adverse health effects from heat events through the use of air conditioning.

**Conclusions:**

Due to the current limited understanding of the full impacts of the wide range of existing climate change adaptation strategies, further research should focus on both unintended positive and negative consequences of public health adaptation.

## Introduction

Climate change is a serious public health threat to people in all countries. Human activities, in particular the burning of fossil fuels, have been identified as key drivers of the climatic changes observed since the mid-twentieth century (Hegerl et al. 2007). The effects of these activities are altering the environment on which we depend, and threaten our collective future through very serious effects on human health (Hegerl et al. 2007; WHO 2009; Confalonieri et al. 2007).

A range of health impacts from climate change have been identified, such as increased cardiopulmonary, infectious, allergic, and mental illnesses as well as a host of weather-related injuries (see Table 1) (McMichael et al. 2003; Haines and Patz 2004; Confalonieri et al. 2007; Seguin 2008; Friel et al. 2011). Key health impacts relate to increases in air pollution, water- and food-borne contamination, the spread of disease carrying vectors and rodents and extreme weather events. Some population groups such as seniors, the socially disadvantaged, aboriginal people, the chronically ill and disabled people, are more vulnerable to the health impacts (Seguin 2008). Particular challenges, such as food insecurity, socio-economic dislocation and political instability and conflict, may be faced by developing countries as a result of these changes (Costello et al. 2009).

**Table 1:** Health impacts of climate change (Haines and Patz 2004; Seguin 2008; Friel et al. 2011)

Climate change effects	Examples of related health risks
Temperature changes	Cardiovascular, cerebrovascular, and respiratory disease
Extreme weather, changes in precipitation	Injuries, diarrheal diseases, malnutrition, respiratory infections, depression, anxiety
Air pollution	Respiratory, cardiovascular disease
Pollen production	Allergic diseases
Microbial contamination and transmission	Malaria, dengue fever, schistosomiasis, lyme disease, hantavirus pulmonary syndrome, leptospirosis
Reduced crop yield	Malnutrition
Displaced populations	Poverty, depression, anxiety, malnutrition

Many adaptation strategies to address health risks from climate change have been identified (Confalonieri et al. 2007; Seguin 2008; Cash 2011). We summarized a non-exhaustive list of common adaptation strategies to five key health outcomes identified by public health authorities and researchers—those related to increased air pollution, extreme heat events, water- and food-borne diseases, vector- and rodent-borne diseases, and extreme weather events. See Table 2 for examples of adaptations.

**Table 2:** Examples of adaptation strategies (Confalonieri et al.  2007; Seguin 2008; Keim 2011; Cash 2011)

Health risks	Adaptation strategies
Heat-related	Urban planning and increase in shading Establish hot-weather response plans and early warning systems Buddy system to check on neighbors during heat waves
Extreme weather related	Improve land-use planning and environmental management (i.e., limit development in high-risk areas such as floodplains or coasts, defensive structures to minimize flash floods) Enhance quantitative data on short-term and long-term health impacts of extreme weather events Maintain and improve disaster management programs for local public health facilities to provide rapid health needs
Air pollution related	Provide information to the public about actions to reduce exposure to air pollution, air quality index, emission standards Identify vulnerable populations Increase capacity for hospital care and physician clinics
Food and water-borne disease	Maintain and upgrade water treatment, sewage and sanitation facilities Surveillance of water- and food-borne diseases Make available new drugs and treatments
Vector and rodent-borne disease	Travel, importation and quarantine laws Surveillance of vector populations Develop and make available new vaccines

The World Health Organization (WHO 2009) has called for the adoption of both greenhouse gas (GHG) mitigation measures and adaptation strategies to address climate change. Many specific mitigation and adaptation strategies which reduce the effects of climate change have been identified by international organizations such as the Intergovernmental Panel on Climate change (IPCC) (Confalonieri et al. 2007) and WHO (McMichael et al. 2003), and by national and sub-national health agencies (Seguin 2008; Cash 2011). Potentially large improvements to health maybe attained through well-designed adaptation strategies that either directly reduce health risks or indirectly improve health outcomes through the achievement of health co-benefits (Ebi et al. 2012). Health co-benefits occur when adaptation strategies produce health advantages outside of the scope of the original health outcomes targeted to be improved; conversely, adaptation actions can pose risks to health if not designed and implemented with human health considerations in mind (Ebi et al. 2012). Proper design and implementation of health adaptation strategies can maximize co-benefits and reduce negative consequences.

At the time of writing, there has yet to be a comprehensive review of the research into the health co-benefits and risks of climate change with respect to health adaptation strategies. Existing literature on adaptation costs and benefits is scattered in terms of sectoral and regional coverage, and does not directly address health co-benefits (Adger et al. 2007). The focus of much of the research is on the economic costs of implementing a wide range of adaptation strategies and the expected benefits from addressing specific concerns such as sea-level rise and impacts on coastal areas (Nicholls and Tol 2006; NRTEE 2011). Limited information is available on the costs of public health adaptations (OECD 2008). Methods for estimating the economic costs of implementation and direct benefits to aid in the assessment of adaptation strategies do exist in the literature. However, they do not routinely address the health co-benefits and risks of these actions (Callaway 2004; NRTEE 2011; Ebi et al. 2006).

With a focus on the developed world, this report reviews current literature on health co-benefits and risks to gain a better understanding of how adaptation strategies undertaken by public health professionals may affect the health of the populations they serve. This information can be used in their decision-making processes to prioritize the development and implementation of specific measures in order to protect people from the impacts of climate change. While measures to mitigate climate change through the reduction of GHGs may also result in significant health co-benefits or risks (WHO 2009; Haines et al. 2009; Ebi et al. 2012), the focus of this paper is on adaptation strategies.

## Methods

A literature review was conducted in April 2012 using electronic databases including Medline, Web of Science, and GEOBASE. In addition, relevant websites and materials from the IPCC and WHO were searched for further data. Peer-reviewed literature from January 2000 to March 2012 was retrieved using a standard search strategy based on the key words “climate”, “climate change”, “adaptation”, “health status”, “public policy”, “health co-benefits”, “health risk”, and “public health.” Only publications in English were included. Literature relevant to a developed nation’s context was examined. A total of 812 articles were identified and titles and abstracts were scanned for relevance. Articles that did not directly address the health co-benefits or risks of adaptation strategies were excluded, with 22 peer-reviewed articles remaining. The types of article were not used as inclusion and exclusion criteria, due to the limited number of articles that addressed this topic.

## Results

There are limited numbers of published articles discussing the health co-benefits and risks of public health adaptation strategies. In most articles, health co-benefits and risks of adaptation were addressed in passing and lack detail. No specific studies directly studied the topics of interest. The co-benefits and risks identified in the literature most commonly related to impacts on health associated with actions that increase social capital and improve urban design. We have also discussed more general, transferrable public health measures that help to address the impact of climate change. See Table 3 for a summary of the results.

**Table 3:** Health co-benefits and risks of adaptation strategies available in the climate change literature

	Adaptation strategies that increase social capital	Sources
Health co-benefits	Decreased heat-related illnesses	Naughton et al. (2002)
	Improved overall health status due to increase capital, independent of other predictors of health	D’Hombres et al. (2010), Fujiwara and Kawachi (2008), Giordano and Lindstrom (2010), Lochner et al. (2003), Schultz et al. (2008), Kjellstrom and Weaver (2009), Bambrick et al. (2011), Younger et al. (2008)
Health risks	Misinformation may be spread with increased social capital leading to worse heat adaptability	Wolf et al. (2010)

	Adaptation strategies that influence urban design	Sources
Health co-benefits	Increased physical activity, decreased cardiovascular and respiratory diseases, thermal comfort, improved mental health	Friel et al. (2011), Woodcock et al. (2009), Younger et al. (2008), Harlan and Ruddell 2011, Frumkin and McMichael (2008), Bambrick et al. (2011), Sullivan and Chang (2011), Eigenbrod et al. (2011)
Health risks	Increased allergic diseases, pests, animals, obscured views and increased fear of crime	Carinanos and Casares-Porcel (2011), Escobedo et al. (2011)
	Air conditioning leading to increased GHG emissions and worsening all health effects of climate change	Akbari et al. (2001), Calm (2002)
Indirect effects	Improved public health including better food handling practices, electricity outage preparedness	Kjellstrom and Weaver (2009), Keim (2011), Ebi et al. (2012)

### Health co-benefits from adaptation strategies that increase social capital

A common source of health co-benefits identified in the literature was adaptation strategies to improve social capital as part of efforts to address climate change health risks stemming from an increase in public health emergencies or national disasters (Ebi and Semenza 2008; Kjellstrom and Weaver 2009; Bambrick et al. 2011). “Social capital” refers to “the ability of actors to secure benefits by virtue of membership in social networks or other social structures” (Portes 1998). It can have a positive influence on health outcomes by increasing access to network-based resources such as counseling during and after a weather-related emergency and by enhancing collective action and the enforcement of social norms that support healthy behaviors (Eicher and Kawachi 2011). Evidence suggests that belonging to a social network can have a protective effect against heat-related illness (Naughton et al. 2002). A low level of social capital, or social isolation, is regarded as contributing to the vulnerability of population groups who are excluded from access to resources needed to protect health (Cutter et al. 2003).

Examples of adaptation strategies that increase social capital include the development of “buddy systems” and community outreach initiatives that target vulnerable populations (e.g., seniors, people with chronic illnesses, socially disadvantaged people) to reduce heat-health risks (Seguin 2008). In terms of health co-benefits, social capital is thought to positively influence health, including mental health, independently of other determinants (d’Hombres et al. 2010; Fujiwara and Kawachi 2008; Giordano and Lindstrom 2010; Lochner et al. 2003; Schultz et al. 2008; Younger et al. 2008).

### Risks to health from adaptations that increase social capital

Potential risks stemming from an inadequate understanding of how social capital works were also identified (Pelling and High 2005). In an article by Wolf et al. (2010), the authors argued that strong bonding networks could actually have the effect of exacerbating vulnerability to the health impacts of climate change. For example, social networks may reinforce misperceptions common among older adults about possible health effects of heat waves and this could act as a barrier to adaptation. A survey of older adults in the United Kingdom in 2007 revealed that many respondents did not feel at significant risk from heat waves personally. Ultimately, the authors offer a cautionary note about social capital suggesting that it is “more complex than simply positive for climate change vulnerability” (Wolf et al. 2010). Greater understanding is needed about the most effective ways to employ social capital to reduce climate change health risks.

### Health co-benefits from adaptation strategies to improve urban design and planning

Adaptation strategies designed to decrease extreme heat events and air pollution in cities—both of which are expected to increase with climate change—commonly involve adjustments to urban design and planning. Strategies such as increased or improved shade and green spaces, smarter road design, the development of walkable neighborhoods, the creation and maintenance of bike paths, and improvement to public transportation are suggested in the literature (Akbari et al. 2001; Addy et al. 2004; Frank et al. 2004; Besser and Dannenberg 2005; Younger et al. 2008; Woodcock et al. 2009; Stone et al. 2010). In addition to directly reducing heat stress and cardiorespiratory disease, these adaptations can lead to increased physical activity, cooling spaces (e.g., shaded areas), and social connectivity which result in a range of health co-benefits such as reduced obesity, cardiovascular disease, and improved mental health (Friel et al. 2011; Woodcock et al. 2009; Younger et al. 2008; Harlan and Ruddell 2011; Frumkin and McMichael 2008; Bambrick et al. 2011; Sullivan and Chang, 2011). Green spaces have also been shown to contribute to flood risk mitigation and so can enhance community safety (Eigenbrod et al. 2011). The resulting co-benefits from these adaptation strategies can have both immediate and long-term positive influences on public health and may have significant economic payoff (Grabow et al. 2012).

In addition, strategies to reduce heat stress by addressing the urban heat island effect (e.g., increasing green spaces, reducing heat absorbing concrete or paved surfaces) and to reduce air pollution by redesigning transportation infrastructure (e.g., building more bike paths and smarter road systems) and through better urban planning (e.g., densification, walkable neighborhoods) may also significantly reduce GHG emissions if designed appropriately (WHO 2009; Susca et al. 2011; Haines et al. 2009; Woodcock et al. 2009; Ebi et al. 2012). If broadly implemented, these adaptation actions may also therefore convey long-term health co-benefits by lessening the rate of climate change and its associated health impacts.

### Risks to health from poor urban design and planning

Communities which do not reduce the urban heat island effect through proper urban design and planning may be forced to rely heavily on air conditioning to reduce heat stress. The use of air conditioning to reduce heat-related morbidity and mortality is identified as an important adaptation measure in many heat adaptation plans (Health Canada 2012). However, it can be a significant source of energy consumption in communities during the summer months (Akbari et al. 2001). Air conditioning produces combustion products, including carbon dioxide and nitrous oxide (Calm 2002), when energy systems rely largely upon the burning of fossil fuels. Both of these gases can lead to increased GHG emissions, increased air pollution, and can exacerbate the urban heat island effect (Calm 2002). For this reason air conditioning should be supplemented with other measures to provide citizens with relief from the heat (e.g., extended pool hours, shaded areas) or to reduce temperatures in urban environments (e.g., UHI mitigation measures) (Health Canada 2012).

As noted above, increasing green spaces is one of the commonly suggested adaptation strategies for reducing the urban heat island. However, in a recent article by Carinanos and Casares-Porcel (2011), a lack of planning in the design of green spaces in urban areas was noted as a potential trigger for pollen allergies. In addition, Escobedo et al. (2011) argues that in the specific case of urban forests, there are costs to the economy and society that must be weighed against the benefits. These might include allergens, pests, insects, leaf litter, debris, falling branches, wild animal droppings and bites, obscured views and increased fear of crime (Escobedo et al. 2011).

### Indirect health co-benefits through increased public health adaptation to climate change

Indirect benefits to health may also be achieved through adaptations that support or strengthen the resources, infrastructures, information systems and processes that underpin existing public health functions. Ebi et al. (2012) suggest that because of the current adaptation deficit and associated impacts on health in most countries, climate change-related investments to prepare the health sector can help address health outcomes not directly attributed to a warming world. This involves the transferability of adaptation strategies for climate change to other public health threats (Kjellstrom and Weaver 2009; Keim 2011). In an examination of international climate change and health adaptation frameworks Clarke and Berry (2012) reported that existing public health risk management standards in the province of Ontario, Canada, were able to address climate change health risks, provided data about impacts to inform interventions. Therefore, the benefits of climate change and health adaptation strategy development are often directly transferable to existing community and regional activities and processes to improve population health. These public health functions constitute the core interests for public health agencies at different levels—population health assessment, health surveillance, health promotion, health protection, disease and injury prevention (Frank et al. 2011; Frumkin et al. 2008). By adapting to climate change impacts, communities will improve their public health systems and public health infrastructure, which will have knock-on positive effects on public health capacity and on the delivery of services and programs to the public. Their development is not only beneficial to the health of the public, but also essential.

## Discussion

There are few published articles examining the health co-benefits and risks of public health adaptation strategies. No comprehensive list of adaptations currently exists that ranks or quantifies potential health co-benefits or risks; consequently, there is little information that is easily accessible for decision-makers to use when identifying priority measures to protect health from climate change impacts. The two examples of specific adaptations discussed here—increasing social capital to reduce health risks from natural hazards and improving urban design to address air pollution and to reduce heat stress—suggest that improvements to health can be achieved through the realization of co-benefits. Increasing social capital to reduce the health impacts of one type of natural hazard (e.g., heat stress) may convey significant health co-benefits associated with protection from other types of hazards. Adaptation strategies designed to decrease extreme heat events and air pollution in cities can additionally lead to increased physical activity, cooling spaces (e.g., shaded areas), and social connectivity, and in turn, contribute to reductions in obesity, cardiovascular disease, and improve mental health.

Examination of public health adaptations that could result in sizeable co-benefits also revealed that care needs to be taken when developing and implementing actions to not harm health. Some measures may inadvertently contribute to increasing the rate of climate change (e.g., wide spread use of air conditioning) or exacerbate the impacts of climate change (e.g., poor design of urban green spaces). They should be examined closely for potential benefits and risks so that the measures adopted will maximize positive health outcomes.

The health co-benefits and possible risks of both *adaptation* and *GHG* *mitigation* measures need to be taken into consideration by decision-makers when addressing climate change. Climate change GHG mitigation strategies prompt a rethinking of lifestyles, energy choices, and consumption patterns which can have substantial health co-benefits associated with urban land transport (Woodcock et al. 2009), household energy (Wilkinson et al. 2009), food and agriculture (Friel et al. 2009), and low-carbon electricity generation (Markandya et al. 2009). Similar to adaptation, GHG mitigation can present a valuable opportunity to achieve substantial health co-benefits (Cifuentes et al. 2001; Aunan et al. 2006; Haines et al. 2009; Kjellstrom and Weaver 2009; Frumkin and McMichael 2008). The WHO reports that “Many policies and individual choices have the potential both to reduce greenhouse gas emissions and produce major health co-benefits…These local and immediate benefits can offset a large part of the costs of climate change mitigation, and provide a strong political and personal motivation for action.” (WHO 2009, p. 3). However, significant differences can exist in the health benefits produced. For example, the adoption of active transport and rapid transit/public transit which is based upon improved land use results in greater immediate health gains than improving fuel efficiency in vehicles (WHO 2011). Therefore, the awareness of such opportunities and information to guide decision-making is crucial for the public health community to optimize their effects (Frumkin and McMichael 2008; Huang et al. 2011).

Lastly, the international community has recognized that the effects of climate change will be differentially experienced by countries around the world. For this paper, we have chosen to focus our discussion on considerations relevant to developed countries. Nonetheless, some adaptation strategies—particularly those that build public health and health system capacity—will be transferrable to low-middle income countries (LMICs). The international community has noted the importance of adaptation strategies for LMICs (Ciscar et al. 2011). The December 2009 Copenhagen Accord (2009) establishes that by 2020, higher income countries (HICs) will provide $100 (US Dollars) billion annually to address the needs of developing countries. This will include funding for adaptation, and efforts to achieve GHG mitigation and temperature targets (Copenhagen Accord 2009). Since adaptation co-benefits often accrue to local populations, adaptation strategies that are common to HICs and LMICs may have differential co-benefits and risks (Callaway 2004). We theorize that the potential co-benefits and risks associated with public health adaptations to climate change may be magnified in LMICs. This will need to be investigated with studies focused on the LMICs.

### Conclusions

There are a limited number of articles addressing the health co-benefits and risks of climate change adaptation strategies. Through this review, we have found health co-benefits and related risks from increases in social capital, and changes in urban design strategies. We have also discussed more general, transferrable public health measures that help to address the impact of climate change; these methods are often core functions of public health. Future research should focus on both short- and long-term, region-specific, positive and negative consequences of adaptation. Liberal use of existing literature in related fields will enhance our understanding of adaptation. Only then can public health practitioners at every level be informed of the full range of health impacts when planning adaptation measures.
